# The SPLUNC1-βENaC complex prevents *Burkholderia cenocepacia* invasion in normal airway epithelia

**DOI:** 10.1186/s12931-020-01454-5

**Published:** 2020-07-17

**Authors:** Saira Ahmad, Christine Seul Ki Kim, Robert Tarran

**Affiliations:** 1 Department of Cell Biology and Physiology, The University of North Carolina, Marsico Lung Insitute, 115 Mason Farm Rd CB 7545, UNC, Chapel Hill, NC 27599 USA; 2 Present address: Max Planck Institute for Biology of Ageing, Cologne, Germany

**Keywords:** CFTR, BPIFA1, pH, Cystic fibrosis, Innate defense

## Abstract

Cystic fibrosis (CF) patients are extremely vulnerable to *Burkholderia cepacia* complex (Bcc) infections. However, the underlying etiology is poorly understood. We tested the hypothesis that short palate lung and nasal epithelial clone 1 (SPLUNC1)–epithelial sodium channel (ENaC) interactions at the plasma membrane are required to reduce Bcc burden in normal airways. To determine if SPLUNC1 was needed to reduce Bcc burden in the airways, SPLUNC1 knockout mice and their wild-type littermates were infected with *B. cenocepacia* strain J2315. SPLUNC1 knockout mice had increased bacterial burden in the lungs compared to wild-type littermate mice. SPLUNC1-knockdown primary human bronchial epithelia (HBECs) were incubated with J2315, which resulted in increased bacterial burden compared to non-transduced HBECs. We next determined the interaction of the SPLUNC1-ENaC complex during J2315 infection. SPLUNC1 remained at the apical plasma membrane of normal HBECs but less was present at the apical plasma membrane of CF HBECs. Additionally, SPLUNC1-βENaC complexes reduced intracellular J2315 burden. Our data indicate that (i) secreted SPLUNC1 is required to reduce J2315 burden in the airways and (ii) its interaction with ENaC prevents cellular invasion of J2315.

## Background

Cystic fibrosis (CF) is a genetic, multi-organ disease that results from loss in function of the CF transmembrane conductance regulator (CFTR)-anion channel. Subsequently, diminished anion secretion results in dehydration and loss of pathogen clearance, causing an increase in chronic bacterial infections and inflammation in the lower airways, and eventual reduction in lung function [1, 2]. Indeed, over 80% of the CF population ultimately succumbs to respiratory failure [3]. Respiratory infections start early in life, with *Staphylococcus aureus* and *Haemophilus influenza* predominantly colonizing the lungs of CF children, and *Pseudomonas aeruginosa* colonization occurring in older children and teenagers [4]. The *Burkholderia cepacia* complex (Bcc), which consists of 18 genetically distinct Gram-negative species and is relatively harmless in normal individuals, but especially virulent in CF patients [5–8]. The epidemic *B. cenocepacia* lineage ET-12 (strain J2315), is predominantly found in European and Canadian CF populations [6]. Rather than colonizing the airways, it leads to cellular invasion and septicemia called Cepacia syndrome that is associated with a rapid decline in health and significantly increased mortality [9, 10]. Moreover, J2315 are multidrug resistant, making treatment difficult [1, 11, 12] and CF patients with J2315 infections are usually not considered for lung transplants as survival rates after transplantation are low [13].

The short palate, lung, and nasal epithelial clone (SPLUNC1; gene name BPIFA1; also referred to as PLUNC, SPURT, NASG, and LUNX) is found in human saliva, sputum, nasal fluid, and bronchoalveolar lavage (BAL) at concentrations of up to 10 μM [14, 15]. SPLUNC1 shares structural homology to the bactericidal/permeability-increasing (BPI) proteins and increases in concentration during Th1 inflammation. SPLUNC1 has surfactant-like and antimicrobial-activity against Gram-negative bacteria, such as *P. aeruginosa, Klebsiella pneumonaie,* and J2315 [15–21]. SPLUNC1 knockout mice infected with *P. aeruginosa* are unable to clear bacterial infections [17]. Additionally, SPLUNC1 maintains airway hydration through negative regulation of the epithelial sodium channel (ENaC). Knockdown of SPLUNC1 in human bronchial epithelial cultures (HBECs) leads to airway surface liquid (ASL) dehydration and in chinchilla models decreases mucociliary clearance [22, 23]. We previously solved the crystal structure of SPLUNC1 and found that it possesses salt bridges that enables it to interact with ENaC in a pH-dependent fashion [19]. These charged surfaces also maintain antimicrobial activity in a pH-dependent manner [24]. Importantly, SPLUNC1 fails to bind to the apical surface of CF HBECs at acidic pH (≤ pH 7) and its failure to bind to ENaC at the acidic ASL pH contributes to Na^+^ hyperabsorption and airway surface liquid dehydration in CF airways [19]. As such, incapability of the CF lung’s innate defense system leads to chronic colonization with opportunistic bacteria [25].

In normal airways, SPLUNC1 binds to the β-subunit of ENaC. While the αγENaC become internalized, βENaC remains at the apical membrane of HBECs and bound to SPLUNC1 to form a SPLUNC1-βENaC complex [26]. SPLUNC1 has anti-J2315 effects in cell-free systems [20] but its relevance in vivo, and to CF pathogenesis remains unclear. Here, we further explored the physiological role of the cell surface SPLUNC1-βENaC complex and we tested the hypothesis that a failure of SPLUNC1-ENaC interactions had adverse consequences on innate defense in the lung against J2315 infections.

## Materials and methods

### Bacterial culture

*B. cenocepacia* strain J2315 was grown in Luria broth (LB) as described [20].

### Expression and purification of human SPLUNC1

Recombinant human SPLUNC1 (rSPLUNC1) were expressed and purified as previously described [19, 27]. SPLUNC1 tagged with either AlexaFluor 594 or 633 were made using Dylight NHS Ester Dyes (Thermo Scientific) following the manufacturer’s protocol.

### Constructs

Human wild type α-, β-, γ-ENaC were used in combinations of tagged subunits as previously described [26].

### Tissue procurement and cell culture

Normal and CF HBECs were obtained from main stem bronchi following protocols approved by UNC’s Office of Human Research Ethics and cultured as described [28]. Demographics of bronchi donors are listed in Table 1. Knockdown of SPLUNC1 was performed using lentiviral shRNA as described [23]. Human embryonic kidney (HEK293T) cells were cultured in MEM-α media with 10% FBS, 1x penicillin/streptomycin at 37 °C, 5% CO_2_.

**Table 1:** Donor demographics for normal and CF bronchi. Mean ± SD ages were 38.9 ± 13.4 and 24.6 ± 8.0 years for normal and CF donors respectively

	Donor	Age	Sex	Ethnicity	Diagnosis/Genotype
**Normal bronchi donors**	**1**	46	F	Caucasian	N/A
	**2**	55	F	Caucasian	N/A
	**3**	43	M	Caucasian	N/A
	**4**	22	F	Caucasian	N/A
	**5**	54	F	Caucasian	N/A
	**6**	46	F	Caucasian	N/A
	**7**	36	M	Unknown	N/A
	**8**	37	M	African American	N/A
	**9**	31	F	Caucasian	N/A
	**10**	16	M	Caucasian	N/A
	**11**	52	M	Caucasian	N/A
	**15**	60	M	Caucasian	N/A
**CF bronchi donors**	**1**	29	F	Caucasian	delF508/1154 ins TC
	**2**	37	M	Unknown	delF508/delF508
	**3**	28	F	Caucasian	delF508/R347P
	**4**	27	F	Unknown	delF508/delF508
	**5**	23	F	Unknown	delF508/W1282X
	**6**	30	F	Unknown	del508/3905insT
	**7**	28	M	Unknown	delF508/delF508

### Mouse infection

Studies were conducted in accordance with NIH guidelines and approved by the Institutional Animal Care and Use Committee at UNC-Chapel Hill. Eight to ten week of age C57BL/6 J SPLUNC1^−/−^ and wild-type littermate mice were infected through intratracheal instillation with 5 × 10^7^ colony forming units (CFU)/mouse J2315, and sacrificed 24 h later as previously described [17]. BAL was collected and whole lung was excised and homogenized to determine bacterial counts.

### Cell infection

For HBECs, cells were washed apically to remove endogenous SPLUNC1, pretreated ±10 μM rSPLUNC1 and inoculated apically with 10^3^–10^6^ CFU/ml J2315 (in log phase growth) for 2 h, 37 °C. For HEK293T cells were transfected using Lipofectamine 2000 (Invitrogen) following the manufacturer’s instructions and transfected with 0.5 μg/DNA of each construct 12 h before the experiment. HEK293T cells were infected with J2315 at MOI of 30 for 2 h, 37 °C.. For HBECs, 100 μl PBS was added apically for 5 min and then collected as apical lavages. HBECs were then washed 5x with PBS before from HBECs before cell lysis. Media/apical lavages and cell lysates for both HEK293T cells and HBECs were collected and aliquots were serially diluted and plated on LB agar plates to determine bacterial counts.

### Cell imaging

HBECs were incubated with 10 μM rSPLUNC1 labeled with DyLight633 and 0.1 μM calcein-AM (green) mucosally for 3 h, 37 °C, 5%CO_2_. Cells were then washed and ASL was labeled with dextran-rhodamine. Transfected HEK293T cells were pretreated with rSPLUNC1 for 1 h, 37 °C, and infected with J2315 labeled with Syto9 (Thermo) at MOI 30 for 2 h, 37 °C [29]. HEK293T cells were labeled with SNARF-1 30 min before imaging.

### Endogenous SPLUNC1 detection

10 μg of sample was denatured and probed for a western blot as previously described [27].

### Statistical analysis

All data are shown as mean ± standard deviation. Data were analyzed using software Prism (GraphPad Software, Inc.) using either Lorentizian curve fitting, nonparametric tests (Kruskal-Wallis followed by Dunn’s multiple comparison test, or the Mann-Whitney U-test, as appropriate). *p* < 0.05 was considered statistically significant. All experiments were performed a minimum of three separate times.

## Results

### The absence of SPLUNC1 increases bacterial susceptibility in murine airways and in HBECs

**Fig. 1:**
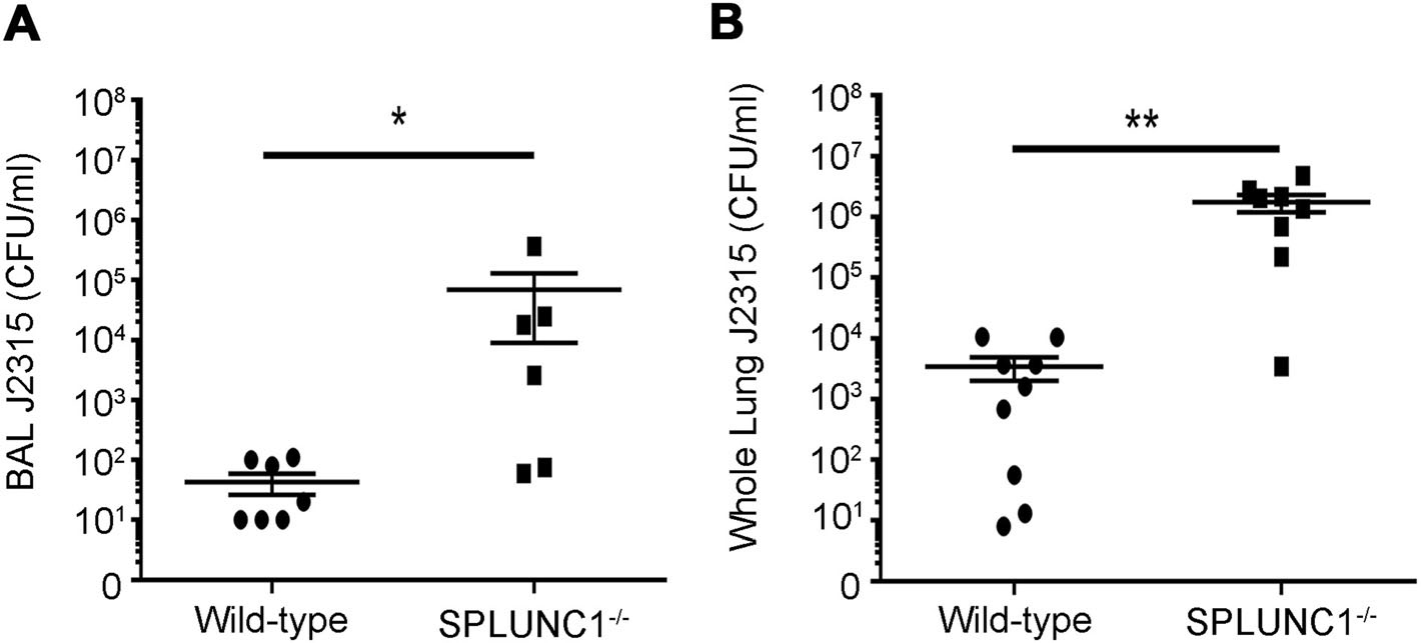
SPLUNC1 knockout mice have increased susceptibility to J2315 infection. Male and female SPLUNC1^−/−^ and wild-type littermate mice were infected intratracheally with 5 × 10^7^ CFU/mouse J2315. 24 h after infection, mice were euthanized and (**a**) BAL and (**b**) whole lung were collected. Whole lung was homogenized and aliquots of lung and BAL were serially diluted and plated to determine bacterial counts. *N* = 6–8 mice per group. * = *p* < 0.05, ** = *p* < 0.01

J2315, is susceptible to SPLUNC1 in vitro. However, SPLUNC1’s role in immunity against J2315 is poorly understood. Therefore, we focused on SPLUNC1’s antimicrobial activity role against J2315 in the airways. SPLUNC1 knockout (SPLUNC1^−/−^) C57bBL/6 J mice were used as an in vivo model. Male and female SPLUNC1^−/−^ mice and their wild-type littermate controls (SPLUNC1^+/+^) were infected intratreacheally with 5 × 10^7^ CFU/mouse J2315 and the bacterial burden determined 24 h later [17]. Wild-type mice had ~ 30 CFU/ml J2315 in their BAL and ~ 3000 CFU/ml in whole lung lysate (Fig. 1a-b). In contrast, SPLUNC1^−/−^ mice had increased bacterial load (> 3 log_10_ units of J2315) in both the BAL and whole lung compared to wild-type mice, changes that were significantly different (Fig. 1a-b), suggesting that SPLUNC1 is required to reduce J2315 susceptibility in vivo.

**Fig. 2:**
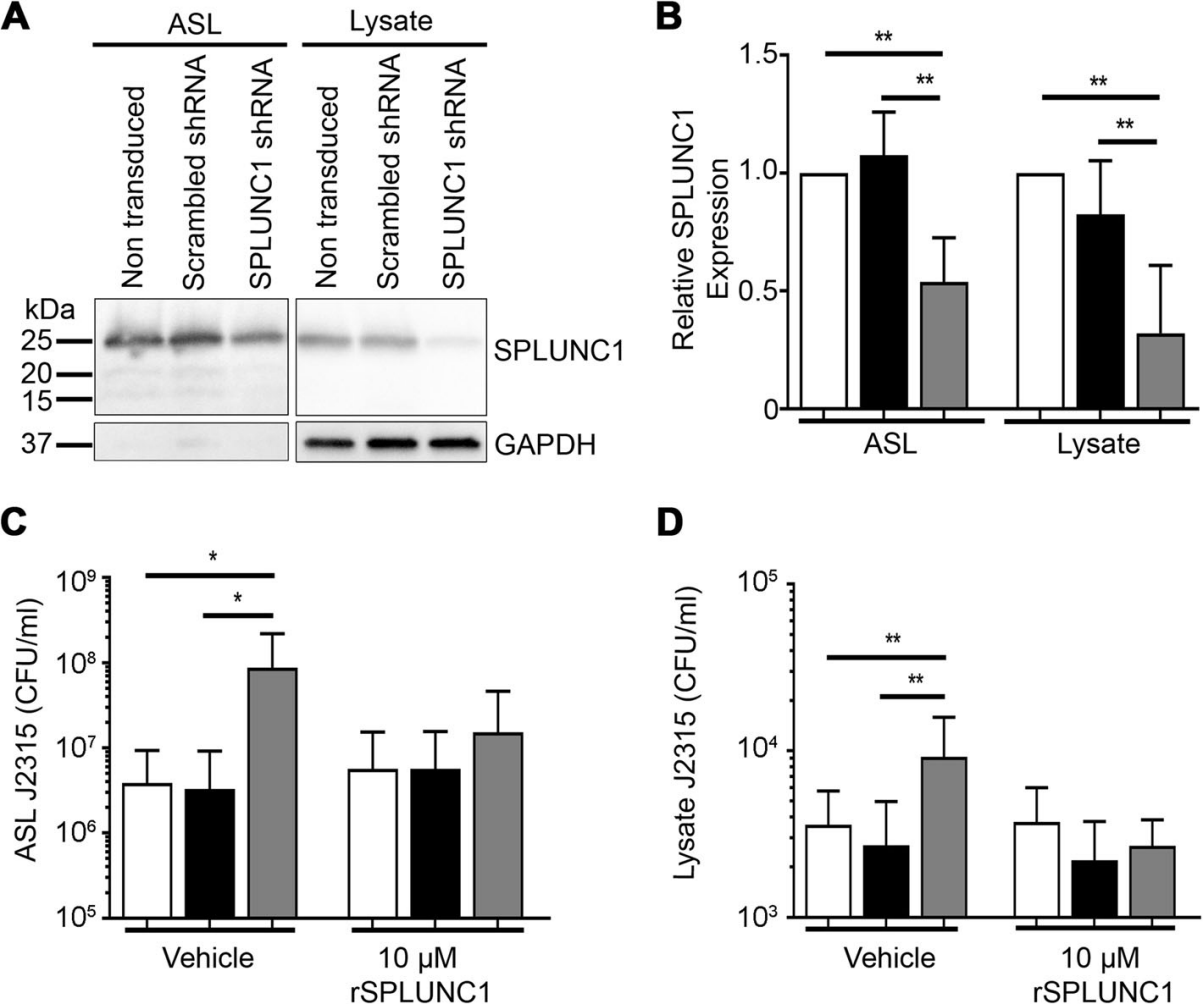
Stable SPLUNC1 knockdown increases J2315 burden in HBECs. SPLUNC1 was stably knocked down in normal HBECs using lentivirus-induced shRNA. Scrambled shRNA was used as a control. **a** Western blots of SPLUNC1 in ASL and in whole cell lysate. **b** Mean densitometry, non-transduced (white bar), scrambled shRNA (black bar), SPLUNC1 shRNA (gray bar). HBECs were pretreated ±10 μM rSPLUNC1for 1 h, then mucosally infected with 10^6^ CFU/ml J2315 for 2 h, 37 °C, 5% CO_2_. Aliquots of (**c**) ASL lavage and (**d**) cell lysate were serially diluted and plated on agar for bacterial counts. *n* = 2 HBECs cultured from *N* = 4 individual donors. (White bar = non-transduced, black bar = scrambled shRNA, gray bar = SPLUNC1 shRNA). * = *p* < 0.05, ** = *p* < 0.01

Since SPLUNC1 was required to reduce J2315 burden in mice, we next determined SPLUNC1’s antimicrobial role in HBECs from normal donors. We stably knocked down SPLUNC1 in HBECs by lentiviral infection using shRNA. SPLUNC1-knockdown HBECs had reduced SPLUNC1 protein expression in both the ASL and lysate, while scrambled shRNA (control) was without effect (Fig. 2a-b). 10^6^ CFU/ml J2315 was then incubated mucosally for 2 h. Knockdown HBECs (lacking SPLUNC1) showed significantly increased J2315 burden in both the ASL and HBEC lysate (Fig. 2c-d), an effect that was abolished by the addition of 10 μM recombinant SPLUNC1 (rSPLUNC1).

### SPLUNC1’s binding to βENaC protects cells from J2315 invasion

**Fig. 3:**
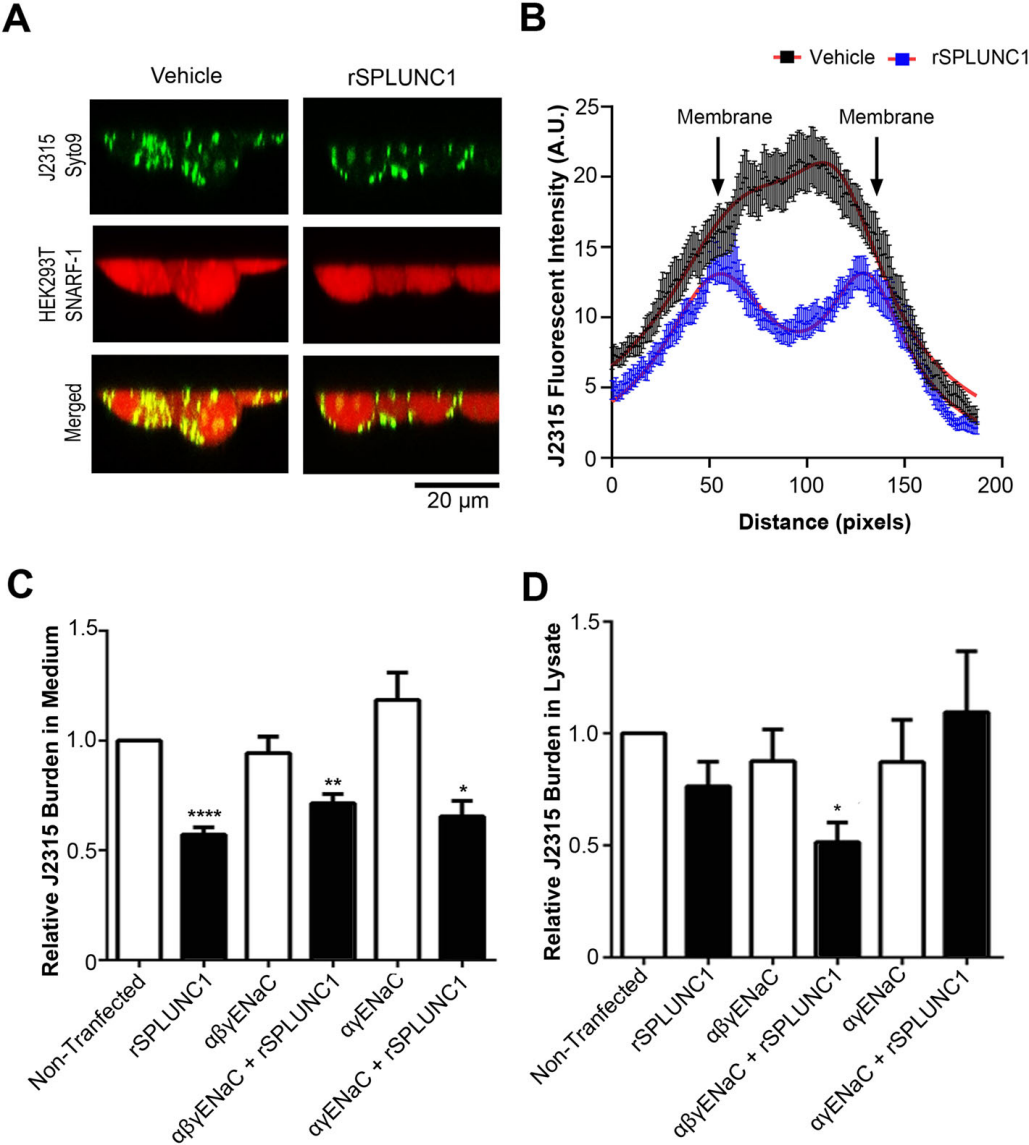
The plasma membrane SPLUNC1-βENaC complex protects cells from *B. cenocepacia* invasion. αβγENaC-transfected HEK293T cells were pre-treated with rSPLUNC1 or vehicle for 1 h, followed by infection J2315 labeled with Syto9 for 1 h at an MOI of 30. Representative XZ (**a**) scanned fluorescence images of *B. cenocepacia*-Syto9 (green) and HEK293T labeled with SNARF-1 (red) (*N* = 3). **b** Graph of mean pixel intensity of J2315 in relationship to cell. Cells pre-treated with vehicle (black) or with rSPLUNC1 (blue) and curve fitted using Lorentizian (*N* = 36), *p* = 0.0001. **c-d** SPLUNC1 was co-expressed with different combinations of ENaC subunits and infected with J2315 for 2 h at MOI of 30 (*N* = 4). Relative J2315 burden in the (**c**) media and (**d**) lysate. * *p* < 0.05, ** *p* < 0.01, **** *p* < 0.0001. Scale bars are 20 μm

We have previously shown that SPLUNC1 preferentially associates with plasma membrane βENaC at the expense of αγENaC [26]. Since βENaC alone is incapable of conducting Na^+^ [30, 31], we next searched for a physiological role for the SPLUNC1-βENaC complex. We hypothesized that the βENaC-SPLUNC1 complex at the plasma membrane formed an antimicrobial shield that limited invasive J2315 from entering cells. SPLUNC1 is present in the ASL at concentrations above the IC_50_ for J2315 (~ 0.1 μM) [20, 32]. We transfected HEK293T cells with αβγENaC or vehicle and pretreated them with either 10 μM rSPLUNC1 or vehicle and infected them with J2315 labeled with Syto9 at a mid-range MOI of 30 for 2 h. HEK293T cells were labeled with SNARF-1. Using confocal microscopy and measuring pixel fluorescent intensity of J2315-Syto9 across the cell, we observed that J2315-Syto9 remained at the cell periphery when pre-treated with rSPLUNC1. However, in the absence of rSPLUNC1, J2315-Syto9 was internalized (*p* = 0.0001) (Fig. 3a-b).

To further study βENaC’s role in preventing J2315 invasion, HEK293T cells transfected with various ENaC subunit combinations ± SPLUNC1 were infected with J2315 for 2 h. In the presence of rSPLUNC1, J2315 levels in the media were significantly reduced, independently of ENaC expression (Fig. 3c). Interestingly, intracellular J2315 levels were only reduced when αβγENaC were co-expressed and rSPLUNC1 was present. However, cells lacking βENaC while in the presence of rSPLUNC1 failed to reduce J2315 internalization (Fig. 3d), suggesting that SPLUNC1’s interaction with βENaC prevents J2315 entry into the cells.

### SPLUNC1 does not reduce bacterial burden in CF HBECs

**Fig. 4:**
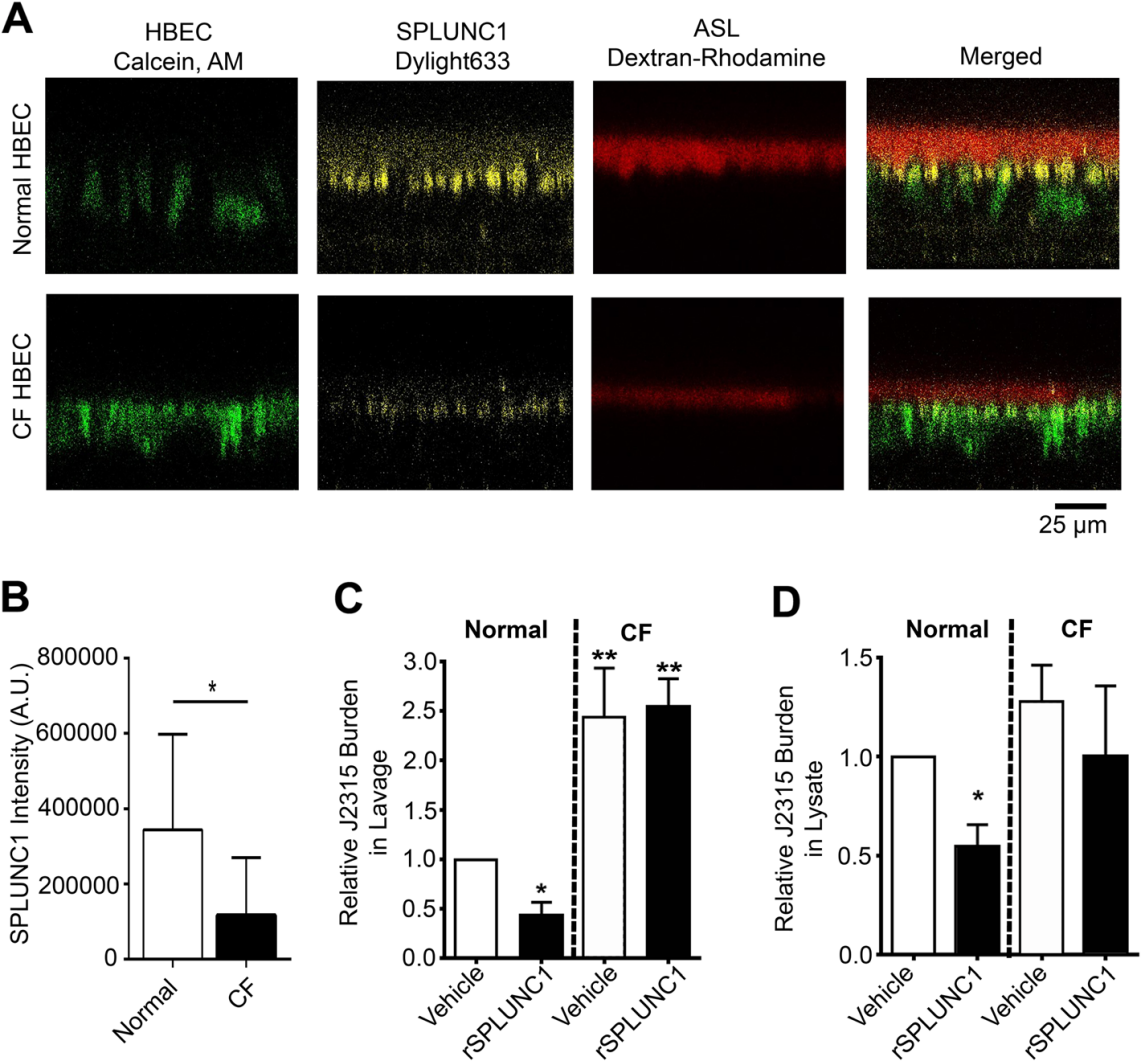
CF HBECs have reduced SPLUNC1 at plasma membrane and increased J2315 burden. Normal and CF HBECs were pre-treated with 10 μM rSPLUNC1-DyLight633 for 3 h. **a** Representative XZ-scanned fluorescence images of HBECs (green), rSPLUNC1-DyLight633 (yellow) and ASL (red) (*N* = 3–5). **b** SPLUNC1 fluorescent intensity from panel A. Normal or CF HBECs were treated with 10 μM rSPLUNC1 (black) or without (white) prior to J2315 infection for 2 h at an MOI of 3 (*N* = 4). Relative J2315 burden in the (**c**) apical lavage and (**d**) cell lysate compared to normal vehicle treatment. All data shown as mean ± SEM. * *p* < 0.05, ** *p* < 0.01. Scale bars are 25 μm

We have previously shown that SPLUNC1 does not bind to ENaC expressed in CF HBECs and fails to maintain antimicrobial activity due to the low pH seen in CF airway surface liquid [19, 24]. We next tested whether the SPLUNC1-βENaC complex provided the same protection against cellular invasion by J2315 in CF human airway epithelia. Normal and CF HBECs were pretreated with 10 μM rSPLUNC1 labeled with DyLight633 and the ASL was labeled with rhodamine-dextran. We have previously shown that SPLUNC1 binding to ENaC is abrogated at the mildly acidic pH seen in CF ASL (~pH 6.5) [19]. Consistent with this observation, SPLUNC1 remained on the apical membrane of normal HBECS but its presence was significantly reduced at CF HBEC apical membranes (Fig. 4a-b) [19]. rSPLUNC1 pretreated HBECs were then infected them with J2315 for 2 h (Fig. 4c-d). rSPLUNC1 decreased J2315 levels in the apical lavage of normal HBECs (Fig. 4c). In contrast, rSPLUNC1 failed to decrease J2315 burden in the lavage of CF HEBCs, suggesting that SPLUNC1’s antimicrobial activity fails in the absence of functional CFTR (Fig. 4d). Moreover, J2315 burden in the lavage was significantly greater in CF HBECs compared to normal HBECs (Fig. 4d), suggesting that the CF environment is permissive for J2315 survival. We also observed that rSPLUNC1 decreased J2315 burden in the lysate of normal HBECs, indicating that the extracellular binding of SPLUNC1 to βENaC was crucial for protection against cellular invasion (Fig. 4d)*.* Importantly, SPLUNC1 was unable to decrease J2315 burden in the lysate of CF HBECs again indicating that SPLUNC1 is ineffective in CF airway epithelia (Fig. 4d).

## Discussion

While relatively harmless to normal people, Bcc are an extremely virulent group of pathogens in immunocompromised patients, including those with CF. Infection with these pathogens often results in Cepacia syndrome, which is characterized by necrotizing pneumonia and septicemia followed by death [8, 10]. Unlike other Gram-negative bacteria (e.g. *P. aeruguinosa*) that tend to chronically colonize CF mucus, Bcc are invasive and rapidly enter the epithelia [8]. Why CF patients are so susceptible to this organism is not known. However, the airways’ innate immune system provides the first line of defense against invading pathogens, and utilizes phagocytic cells, cytokines, mucins and AMPs to prevent bacterial colonization [33, 34]. Whilst AMPs such as LL-37 and human beta defensins have been extensively studied [12, 35], less is known about SPLUNC1’s antimicrobial abilities. However, here we demonstrated that SPLUNC1 played a hitherto underestimated role in preventing J2315 infection. Mice lacking SPLUNC1 (SPLUNC1^−/−^) were significantly more susceptible to J2315 with increased bacterial growth than their wild-type littermate controls (Fig. 1), which is consistent with previous reports of increased susceptibility to *P. aeruginosa* and *K. pneumonaie* [16, 17]. Additionally, stable knockdown of SPLUNC1 expression in normal HBECs similarly resulted in increased J2315 burden, while addition of rSPLUNC1 to the ASL reduced J2315 levels, suggesting that in vivo effects of SPLUNC1 on J2315 are reprised in vitro (Fig. 2). As part of the innate immunity, SPLUNC1 also increases mucocilliary clearance that can reduce bacterial burden in the airways [15, 17, 21]. Indeed, mice that overexpress SPLUNC1 have increased protection against *Mycoplasma pneumonia* and *P. aeruginosa* [36, 37]. Given the extent of J2315 burden in SPLUNC1^−/−^ mice and SPLUNC1 knockdown HBECs, we propose that SPLUNC1, therefore likely plays a pivotal role in preventing J2315 infection in mammalian airways.

Consistent with recent studies on the effect of SPLUNC1 on J2315 [20, 21, 24], our data indicated that SPLUNC1 limited J2315 burden in the extracellular milieu independently of ENaC expression levels. However, SPLUNC1 in the absence of βENaC failed to limit J2315 invasion (Fig. 3). Thus, the interaction between βENaC and SPLUNC1 at the plasma membrane appeared necessary to reduce J2315 invasion. SPLUNC1 is a multifunctional protein, which has different domains that perform different functions. Its S18 domain interacts with ENaC [38], the α6 domain interacts with the Orai1 Ca^2+^ channel, while the α4 domain contains its antimicrobial activity [21, 24, 39]. We have previously shown that αβγENaC dissociates once SPLUNC1 binds to βENaC, resulting in αγENaC being internalized while βENaC remains at the plasma membrane bound to SPLUNC1 [26]. J2315 interacts with epithelial cells’ glycolipid receptors via its cable pili [40] resulting in intracellular invasion through membrane-bound vacuoles or rearrangement of cytoskeleton [41, 42]. However, SPLUNC1 is abundantly found in the airways and can bind to the lipopolysaccharide (LPS) of Gram-negative bacterial membranes [18] to inhibit bacterial adhesion, and endocytosis into epithelial cells. Indeed, inhibition of J2315 attachment to epithelial cells by dextran resulted in reduced virulence in the lungs [43]. We therefore speculate that αγENaC served as a chaperone to bring βENaC to the plasma membrane, where it is presented to SPLUNC1, which simultaneously serves as an antimicrobial shield to prevent bacterial internalization.

Despite being effective in normal HBECs, SPLUNC1 failed to affect J2315 burden in the airway surface liquid of CF HBECs and was also ineffective at reducing cellular invasion into CF cultures (Fig. 4). SPLUNC1 contains salt bridges that are pH-sensitive, which renders SPLUNC1 nonfunctional in acidic conditions such as that seen in CF HBECs. Non-functional SPLUNC1 is unable to bind to ENaC [1] resulting in less SPLUNC1 at the apical membrane of CF HBECs. In normal conditions, SPLUNC1 binds to βENaC and remains at the plasma membrane [2]. Additionally, SPLUNC1 fails to reduce bacterial burden in the acidic pH of the CF airways [24]. Thus, we hypothesize that the abnormal pH in CF airway surface liquid leads to a double hit in that it (i) directly reduces SPLUNC1’s antimicrobial activity and (ii) prevents SPLUNC1 from binding to ENaC at the apical plasma membrane. These failures of SPLUNC1’s antimicrobial actions, combined with a failure to clear mucus, reduce CF airway epithelia’s ability to prevent J2315 invasions. Similar characteristics have been seen with other antimicrobial proteins: Attacin and collectin will bind to the LPS and remain in the outer membrane of Gram-negative bacteria to reduce bacterial burden [44, 45]. Other peptides, such as CdsN peptide, have been shown to disrupt protein interaction of bacteria’s type III secretion system preventing bacterial invasion [46]. Here SPLUNC1 is able to both i) remain in the bacteria’s outer membrane [18], while ii) prevent invasion.

## Conclusion

Our data indicate that the absence of SPLUNC1-βENaC’s antimicrobial shield leaves CF airway epithelia especially vulnerable to J2315 invasion. This may explain why CF lungs are especially vulnerable to increased cellular invasion and mortality [9, 10]. Thus, we propose that the interaction between SPLUNC1 and ENaC serves the dual function of (i) regulating airway hydration by limiting Na^+^ absorption and (ii) of preventing J2315 invasion. Therapeutic strategies that restore SPLUNC1-βENaC interactions in CF airways, such as raising airway surface liquid pH, may not only limit ENaC hyperactivity but could also reduce levels of bacterial infections by placing SPLUNC1 at the apical plasma membrane.

## Data Availability

All data generated or analyzed during this study are included in this published article.

## Abbreviations

AMP: Antimicrobial peptides
ASL: Airway surface liquid
BAL: Bronchoalveolar lavage
Bcc: *Burkholderia cepacia* complex
BPI: Bactericidal/permeability-increasing
CF: Cystic Fibrosis
CFTR: Cystic fibrosis transmembrane conductance regulator
CFU: Colony forming units
ENaC: Epithelial sodium channel
HBEC: Human bronchial epithelia cell
HEK293T: Human embryonic kidney
LB: Luria broth
SPLUNC1: Short palate lung and nasal epithelial clone 1
rSPLUNC1: Recombinant human short palate lung and nasal epithelial clone 1
